# Supplementary material for: Brainstem noradrenergic modulation of the kisspeptin neuron GnRH pulse generator in mice
Source: Nat Commun. 2025 Jul 1;16:5772. doi: 10.1038/s41467-025-60837-8 (PMC12215617; doi:10.1038/s41467-025-60837-8)
Supplement: Supplementary file 1 — Supplementary Information [file 41467_2025_60837_MOESM1_ESM.pdf]

Supplementary information

Brainstem noradrenergic modulation of the kisspeptin neuron GnRH pulse generator in mice

Szilvia Vas, Paul G. Morris, Zulfiye Gul, Miguel Ruiz-Cruz, Su Young Han, Allan E. Herbison

Department of Physiology, Development and Neuroscience, University of Cambridge, Cambridge CB2 3EG, United Kingdom

*Correspondence should be addressed to Allan E. Herbison (email: aeh36@cam.ac.uk)

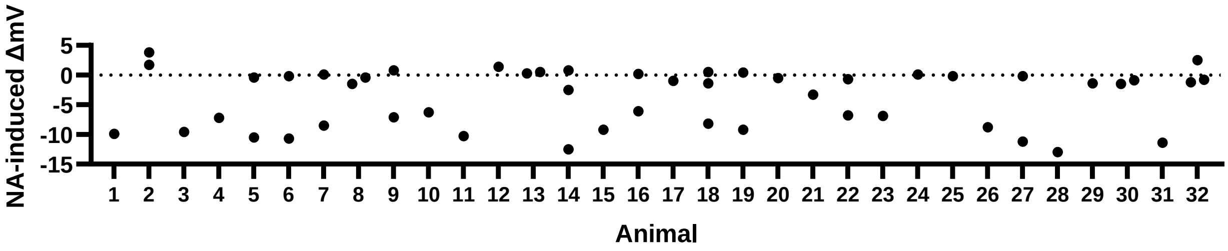

Supplementary Figure 1. Individual kisspeptin neurons exhibit heterogenous hyperpolarizing responses to noradrenaline (NA). Amplitude of membrane voltage change (V_{mem}) in response to NA application for each neuron recorded from each animal. Neurons are grouped by animal, with 1-3 cells recorded per animal. n = 50 neurons from 50 slices taken from 32 animals.

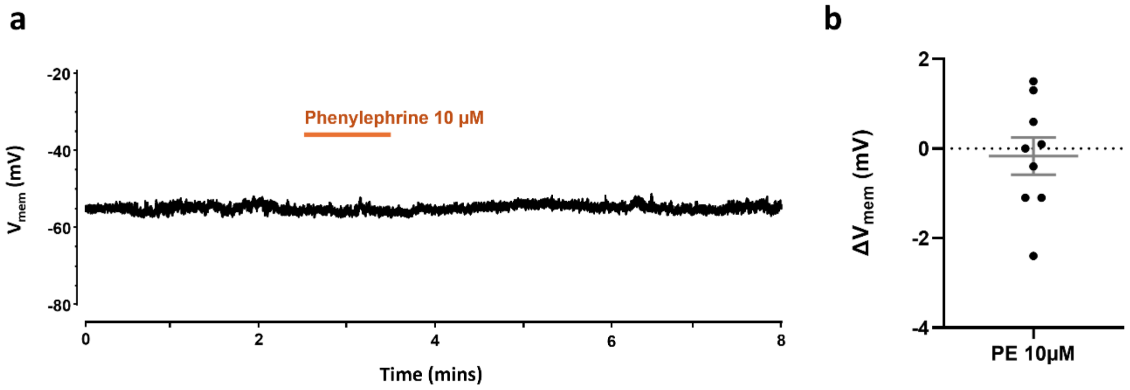

Supplementary Figure 2. ARN^{KISS} neurons do not respond to the α1 agonist phenylephrine. (**a**). Representative whole-cell patch clamp trace from an ARN^{KISS} neuron (in the presence of TTX, CNQX, DAP5, and BIC) showing membrane voltage in response to a 1 min phenylephrine (10 µM) application. (**b**). Change in membrane voltage in the presence phenylephrine (PE) in ARN^{KISS} neurons (n = 9 cells from 9 slices, 5 diestrous female animals).

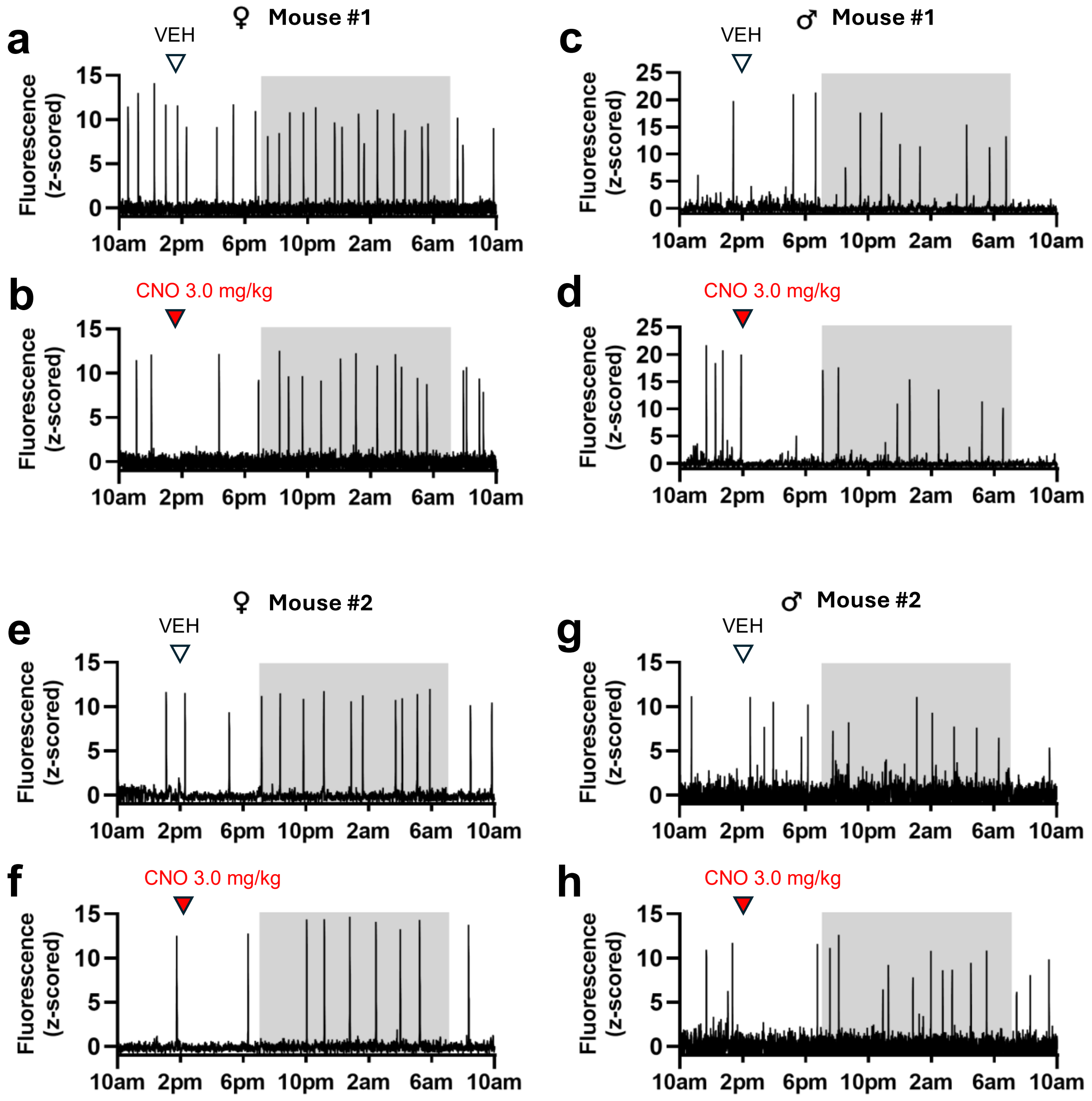

Supplementary Figure 3. Chemogenetic activation of ARN-projecting NA neurons slows ARN^{KISS} neuron population synchronization events. Representative 24-h ARN^{KISS} neuron GCaMP photometry traces from hM3Dq-expressing female and male mice during which ARN-projecting NA neurons were chemogenetically activated by injecting 3 mg/kg clozapine-N-oxide (CNO) 4-h after starting the recording. Each upstroke represents a population synchronization event. Seven male and 7 female replicates were undertaken with similar results (see Fig.4). (**a-b**, **e-f**) Diestrous females #1 and #2 given vehicle (VEH) (**a**,**e**) and CNO (**b**,**f**). (**c-d**, **g-h**). Males #1 and #2 given vehicle (VEH) (**c**,**g**) and CNO (**d**,**h**). The shaded area indicates the period of lights off. Peaks represent ARN^{KISS} neuron population SEs.

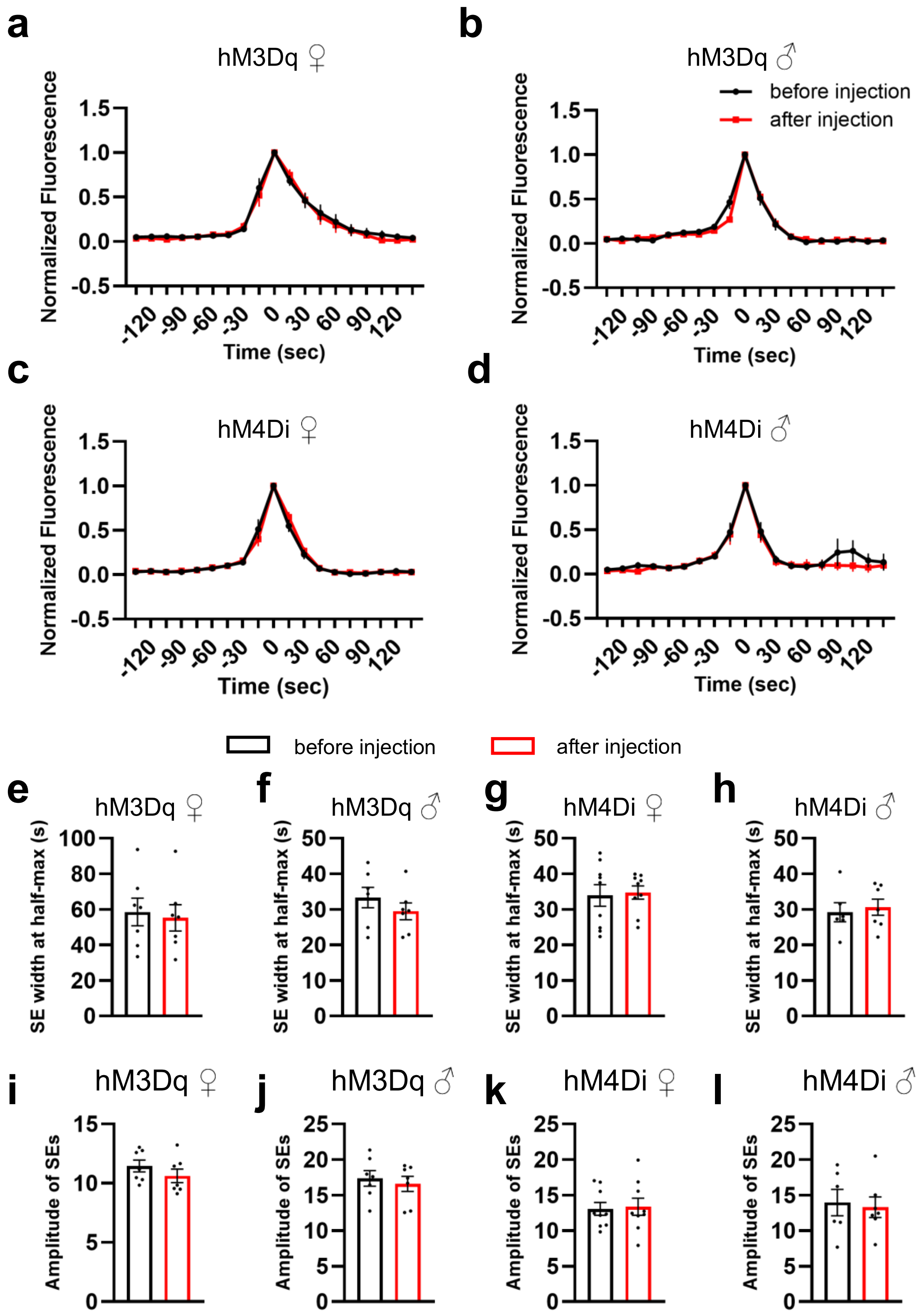
a
hM3Dq ♀
Normalized Fluorescence
Time (sec)
b
hM3Dq ♂
before injection
after injection
c
hM4Di ♀
d
hM4Di ♂
before injection
after injection
e
hM3Dq ♀
SE width at half-max (s)
f
hM3Dq ♂
g
hM4Di ♀
h
hM4Di ♂
i
hM3Dq ♀
Amplitude of SEs
j
hM3Dq ♂
k
hM4Di ♀
l
hM4Di ♂

Supplementary Figure 4. Chemogenetic activation or inhibition of NA inputs to the ARN have no effect on the dynamics of individual ARN^{KISS} neuron synchronization events (SEs). (**a-d**) Line graphs showing the profile of SEs (represented by normalized fluorescence) in hM3Dq- (**a, b**) and hM4Di-expressing (**c, d**) female (**a, c**) and male (**b, d**) mice before (black) and after (red) administration of 3 mg/kg CNO. (**e-h**) Bar graphs showing the mean ± SEM width of SEs of ARN^{KISS} neurons measured at the half of the maximum amplitude in hM3Dq (**e, f**) and hM4Di (**g, h**) -expressing female (**e, g**) and male (**f, h**) mice before and after 3 mg/kg s.c. CNO. (**i-l**) Bar graphs showing the mean ± SEM amplitude of SEs in hM3Dq (**i, j**) and hM4Di receptor (**k, l**) -expressing female (**i, k**) and male (**j, l**) mice before (black) and after (red) the administration of 3 mg/kg CNO. hM3Dq females, N = 7; hM3Dq males, N = 7; hM4Di females, N = 9; hM4Di males N = 7.
