# Supplementary material for: Brainstem noradrenergic modulation of the kisspeptin neuron GnRH pulse generator in mice
Source: Nat Commun. 2025 Jul 1;16:5772. doi: 10.1038/s41467-025-60837-8 (PMC12215617; doi:10.1038/s41467-025-60837-8)
Supplement: Supplementary file 2 — Reporting summary [file 41467_2025_60837_MOESM2_ESM.pdf]

nature portfolio

Corresponding author(s): Allan E. Herbison

Last updated by author(s): 27/5/25

Reporting Summary

Nature Portfolio wishes to improve the reproducibility of the work that we publish. This form provides structure for consistency and transparency in reporting. For further information on Nature Portfolio policies, see our Editorial Policies and the Editorial Policy Checklist.

Statistics

For all statistical analyses, confirm that the following items are present in the figure legend, table legend, main text, or Methods section.

n/a	Confirmed	
☐	☒	The exact sample size (*n*) for each experimental group/condition, given as a discrete number and unit of measurement
☐	☒	A statement on whether measurements were taken from distinct samples or whether the same sample was measured repeatedly
☐	☒	The statistical test(s) used AND whether they are one- or two-sided<br>*Only common tests should be described solely by name; describe more complex techniques in the Methods section.*
☒	☐	A description of all covariates tested
☐	☒	A description of any assumptions or corrections, such as tests of normality and adjustment for multiple comparisons
☐	☒	A full description of the statistical parameters including central tendency (e.g. means) or other basic estimates (e.g. regression coefficient) AND variation (e.g. standard deviation) or associated estimates of uncertainty (e.g. confidence intervals)
☐	☒	For null hypothesis testing, the test statistic (e.g. *F*, *t*, *r*) with confidence intervals, effect sizes, degrees of freedom and *P* value noted<br>*Give P values as exact values whenever suitable.*
☒	☐	For Bayesian analysis, information on the choice of priors and Markov chain Monte Carlo settings
☒	☐	For hierarchical and complex designs, identification of the appropriate level for tests and full reporting of outcomes
☒	☐	Estimates of effect sizes (e.g. Cohen's *d*, Pearson's *r*), indicating how they were calculated

Our web collection on statistics for biologists contains articles on many of the points above.

Software and code

Policy information about availability of computer code

Data collection	Open-source Neuromouse fiber photometry data acquisition system developed by Tussock Innovation and Argotech (New Zealand, https://www.otago.ac.nz/neuroendocrinology/resources/argotech.html). Utilizes Doric (Quebec, Canada) LED drivers and components with National Instrument DAQ board. Fluorescent mages were taken by Olympus cellSens Imaging Software version 2.3.
Data analysis	Matlab 2021a (MatWorks, Inc.). GraphPad Prism 10 software (GraphPad software Inc.)<br>ImageJ software (Schneider, Rasband et al. 2012) (v1.54f)<br>Ex vivo data analysis code (Han and Morris et al. 2023), Zenodo repository (https://doi.org/10.5281/zenodo.7334481)<br>GCaMP fibre photometry analysis code, Zenodo repository (https://doi.org/10.5281/zenodo.15320261)

For manuscripts utilizing custom algorithms or software that are central to the research but not yet described in published literature, software must be made available to editors and reviewers. We strongly encourage code deposition in a community repository (e.g. GitHub). See the Nature Portfolio guidelines for submitting code & software for further information.

Data

Policy information about availability of data

All manuscripts must include a data availability statement. This statement should provide the following information, where applicable:

- Accession codes, unique identifiers, or web links for publicly available datasets
- A description of any restrictions on data availability
- For clinical datasets or third party data, please ensure that the statement adheres to our policy

Source data are provided as a Source Data file. All source data generated in this study have been deposited in Dryad under accession code (XXXX) are also available from the University of Cambridge Apollo Repository.

Research involving human participants, their data, or biological material

Policy information about studies with human participants or human data. See also policy information about sex, gender (identity/presentation), and sexual orientation and race, ethnicity and racism.

Reporting on sex and gender	N/A
Reporting on race, ethnicity, or other socially relevant groupings	N/A
Population characteristics	N/A
Recruitment	N/A
Ethics oversight	N/A

Note that full information on the approval of the study protocol must also be provided in the manuscript.

Field-specific reporting

Please select the one below that is the best fit for your research. If you are not sure, read the appropriate sections before making your selection.

☒ Life sciences ☐ Behavioural & social sciences ☐ Ecological, evolutionary & environmental sciences

For a reference copy of the document with all sections, see nature.com/documents/nr-reporting-summary-flat.pdf

Life sciences study design

All studies must disclose on these points even when the disclosure is negative.

Sample size	Sample sizes were chosen based on our previous publications addressing neuroendocrine regulation of reproduction.
Data exclusions	No data has been excluded from this study.
Replication	All experimental observations were repeated multiple times; all these data were included in the datasets.
Randomization	In in vivo and ex vivo experiments animals were assigned to experimental groups randomly, and treatments/drug applications were performed according to a randomized design.
Blinding	No specific methods were used to blind investigators during performing the experiments to treatments (in vivo) or drug applications (ex vivo) during the experiments themselves, but investigators were blinded to the treatments/drug applications as well as sex of the animals when processing data. All data were analysed using consistent parameters and algorithms.

Reporting for specific materials, systems and methods

We require information from authors about some types of materials, experimental systems and methods used in many studies. Here, indicate whether each material, system or method listed is relevant to your study. If you are not sure if a list item applies to your research, read the appropriate section before selecting a response.

Materials & experimental systems

n/a	Involved in the study	
☐	☒	Antibodies
☒	☐	Eukaryotic cell lines
☒	☐	Palaeontology and archaeology
☐	☒	Animals and other organisms
☒	☐	Clinical data
☒	☐	Dual use research of concern
☒	☐	Plants

Methods

n/a	Involved in the study	
☒	☐	ChIP-seq
☒	☐	Flow cytometry
☒	☐	MRI-based neuroimaging

Antibodies

Antibodies used

polyclonal chicken anti-mCherry primary antibody [Abcam, catalog#Ab205402, RRID: AB_2722769] ,
rabbit anti-TH primary antibody [Chemicon, catalog #Ab152;RRID:AB_390204],
goat anti-chicken biotinylated secondary antibody [Vector Laboratory catalog # BA-9010],
goat anti-rabbit secondary antibody conjugated with Alexa Fluor 488 [Invitrogen, catalog # A11008]

Validation

The chicken anti-mCherry antibody (Abcam, catalog#Ab205402, RRID: AB_2722769) specifically detects mCherry (molecular weight: 26kDa). This antibody has been published in 73 publications (including Berry et al., Nat Communications 2023 Mar 17;14(1):1492; Akter et al. Elife 2023 Nov 14:12:e85751; Chen et al. 2002 Mol Psychiatry Dec;27(12):4843-4860.

The rabbit anti-TH primary antibody [Chemicon/Millipore, catalog #Ab152, RRID:AB_390204] selectively labels a single band at approximately 62kDa corresponding to tyrosine hydroxylase. This antibody has been well characterized and used extensively in different mouse models (including Kim et al. Lab Invest. 2019 Sep;99(9):1389-1399; Morgan et al. Metabolism 2024 Aug:157:155940; Tsetsenis et al. Front Cell Neurosci. 2022 Apr 15:16:887679.

Animals and other research organisms

Policy information about studies involving animals; ARRIVE guidelines recommended for reporting animal research, and Sex and Gender in Research

Laboratory animals

All mice were investigated when adult (older than 10 weeks of age).
129S6Sv/Ev C57BL/6 Kiss1Cre/+mice (Prof. Bill Collede, University of Cambridge, UK (Yeo, Kyle et al. 2016))
Ai162 (TIT2L-GC6s-ICL-tTA2)-D Cre-dependent GCaMP6s line (JAX stock #031562) (Daigle, Madisen et al. 2018)
Dbhtm1.1(flpo)Pjen line (Robertson, Plummer et al. 2013)

Wild animals

The study did not involve wild animals.

Reporting on sex

This study was designed to address the key scientific question in both males and females separately.

Field-collected samples

The study did not involve field-collected samples.

Ethics oversight

All animal experimental protocols were approved by the Animal Welfare and Ethics Board of the University of Cambridge (UK Home Office license P174441DE).

Note that full information on the approval of the study protocol must also be provided in the manuscript.

Plants

Seed stocks

N/A

Novel plant genotypes

N/A

Authentication

N/A
